# Noncoding RNAs responsive to nitric oxide and their protein-coding gene targets shed light on root hair formation in *Arabidopsis thaliana*

**DOI:** 10.3389/fgene.2022.958641

**Published:** 2022-09-27

**Authors:** Camilla Alves Santos, Camila Fernandes Moro, Ione Salgado, Márcia Regina Braga, Marília Gaspar

**Affiliations:** ^1^ Laboratório de Ecofisiologia e Bioquímica de Plantas, Núcleo de Conservação da Biodiversidade, Instituto de Pesquisas Ambientais, São Paulo, SP, Brasil; ^2^ Programa de Pós-Graduação em Biologia Celular e Estrutural, Universidade Estadual de Campinas, Campinas, SP, Brasil

**Keywords:** cell wall modulation, miRNA, lncRNA, ncRNA–PCG interaction, auxin, *A. thaliana*

## Abstract

An overview of the total *Arabidopsis thaliana* transcriptome, described previously by our research group, pointed some noncoding RNA (ncRNA) as participants in the restoration of hair-root phenotype in *A. thaliana rhd6* mutants, leading us to a deeper investigation. A transcriptional gene expression profiling of seedling roots was performed aiming to identify ncRNA responsive to nitric oxide (GSNO) and auxin (IAA), and their involvement in root hair formation in the *rhd6* null mutant. We identified 3,631 ncRNAs, including new ones, in *A. thaliana* and differential expression (DE) analysis between the following: 1) GSNO-treated *rhd6* vs. untreated *rhd6*, 2) IAA-treated *rhd6* vs. untreated *rhd6*, 3) GSNO-treated *rhd6* vs. IAA-treated *rhd6*, and 4) WS-2 vs. untreated *rhd6* detected the greatest number of DE genes in GSNO-treated *rhd6*. We detected hundreds of *in silico* interactions among ncRNA and protein-coding genes (PCGs), highlighting MIR5658 and MIR171 precursors highly upregulated in GSNO-treated *rhd6* and wild type, respectively. Those ncRNA interact with many DE PCGs involved in hormone signaling, cell wall development, transcription factors, and root hair formation, becoming candidate genes in cell wall modulation and restoration of root hair phenotype by GSNO treatment. Our data shed light on how GSNO modulates ncRNA and their PCG targets in *A. thaliana* root hair formation.

## Introduction

Transcriptome studies in eukaryotes revealed that more than 90% of the genome is transcribed, with a diverse set of transcripts corresponding to noncoding RNAs (ncRNAs) (Chekanova et al., 2007; Kapranov et al., 2007). Noncoding RNAs are functional and low protein-coding potential RNA molecules, which can be classified according to their function, location, and length. Depending on their length, ncRNAs can be divided into small ncRNAs (sRNAs) (20–30 nt), which are commonly found as transcriptional and translational regulators, medium-sized ncRNAs (50–200 nt), and also long ncRNAs (lncRNAs) (>200 nt). The last one is usually involved in other processes, such as splicing, gene inactivation, and translational regulation (Wang et al., 2017; Yu et al., 2019).

The advent of high-throughput sequencing technologies has facilitated the identification and characterization of many lncRNAs in plants (Fukuda et al., 2020), such as *Oryza sativa* (Li et al., 2020), *Solanum lycopersicum* (Zhu et al., 2015), and *Arabidopsis thaliana* (Lasky et al., 2014). Recent studies indicated that thousands of lncRNAs are extensively distributed in different regions of the plant genomes, including introns, intergenic regions, natural antisense transcripts (NAT), pseudogenes, and retrotransposons in protein-coding genes (PCGs) (Lee 2012; Wang et al., 2017; Kopp and Mendell 2018). NATs are a specific group of lncRNA which are complementary to PCGs, showing tissue-specific responses to biotic or abiotic stresses (Jannesar et al., 2020; Zhao et al., 2020). Long noncoding RNAs are also involved in translational regulation and post-translational modification, thereby regulating protein phosphorylation, ubiquitination, and acetylation, and modulating tissue gene expression during developmental stages and in response to external stimuli (Kim and Sung 2012; Zhang et al., 2019). There is an increasing number of evidence showing that plant lncRNAs have key roles in genomic imprinting, cell differentiation, epigenetic regulation, and stress responses (Zhang et al., 2011; Di et al., 2014; Liu et al., 2015).

To date, the best-studied ncRNAs in plants are sRNAs, including microRNAs (miRNAs) and nucleolar RNA (snoRNA), which play important roles in transcriptional and post-transcriptional regulation of gene expression (Bardou et al., 2014). Some miRNA precursors are well studied in *A. thaliana*, and their relationship with the nitrogen (N) metabolism is known, such as miR160, miR167, miR171, miR393, miR169, miR826, and miR5090 (Gifford et al., 2008; Wang et al., 2010; Liang et al., 2012). Despite the knowledge achieved regarding N-responsive miRNAs already identified, many still remain uncharacterized.

Nitric oxide (NO) is a gaseous signaling molecule originated mainly from the nitrate metabolism in plants through nitrate/nitrite reductase activities (Salgado et al., 2009; Salgado et al., 2017). NO plays a broad role in the regulation of developmental processes in plants (Mur et al., 2013). We reported previously that NO-donor S-nitrosoglutathione (GSNO), but not auxin (IAA), restored the wild-type root transcriptome profile in *rhd6* (*root hair defective 6*) mutants. NO modulates the expression of a large number of genes related to cell wall composition and metabolism, as well as those encoding ribosomal proteins, DNA and histone-modifying enzymes, and proteins involved in post-translational modification (Moro et al., 2017). A glimpse of the total *A. thaliana* transcriptome described by Moro et al. (2017) suggested that some ncRNA could also be involved in the restoration of wild root hair phenotype, leading us to investigate this hypothesis deeper. Considering that studies reporting NO-responsive ncRNAs in *A. thaliana* are still incipient, here, we performed the mapping of *A. thaliana* RNA-seq reads previously generated by Moro et al. (2017) against the reference genome followed by a *de novo* assembly. After that, we carried out a transcriptional gene expression profiling of *A. thaliana* seedling roots with the purpose of identifying ncRNA genes responsive to GSNO and IAA treatments in the *rhd6* null mutant when compared to the wild type (WS-2). We also investigated the putative interactions between ncRNA–ncRNA and ncRNA–PCG pairs, using the root NO-responsive PCGs for *A. thaliana* described by Moro et al. (2017), aiming to detect PCGs targeted and modulated by those ncRNA.

## Materials and methods

### Sampling

Plant cultivation and treatment were performed by Moro et al. (2017). In brief, seeds of *A. thaliana rhd6* mutant and its respective Wassilewskija (WS-2) wild ecotype, obtained from the Arabidopsis Biological Resource Center (ABRC), were germinated in petri dishes containing nutritive medium with the addition of 1 mM *S*-nitrosoglutathione (GSNO; Enzo Life Sciences), 50 nM indole-3-acetic acid (IAA; Sigma-Aldrich), or deionized water. The culture plates were kept in growth chambers at 22°C under a 12-h photoperiod at a light intensity of 85 μmol m^2^ s^−1^ (μƐ). Roots from 5-day-old WS-2, *rhd6*, and IAA- and GSNO-treated *rhd6* seedlings were collected and immediately frozen in liquid nitrogen for RNA stabilization. For RNA isolation, four biological replicates of each condition/treatment were used, totalizing 16 samples sequenced as described in the study by Moro et al. (2017).

### Sequence data analysis

Paired-end libraries were prepared as described in the TruSeq RNA Sample Prep Protocol (Illumina, San Diego, CA, United States). Indexed DNA libraries were normalized, pooled, and sequenced in the paired-end mode in two lanes using an Illumina HiSeq SQ sequencer. All raw reads are available at the Sequence Read Archive (SRA-NCBI) under the accession number SRP285694 (BioProject PRJNA666227) (Moro et al., 2017). The quality of the raw data generated after sequencing was checked in the FastQC software (version 0.10.1) (http://www.bioinformatics.babraham.ac.uk/projects/fastqc/). The *reads* were filtered for Phred quality (QS) 26 (sequence average) and 30 (sequence edges), and a minimum length of 65 bp, using the SeqyClean package (v.1.9.9) (https://github.com/ibest/seqyclean). This program was also used to remove contaminant sequences from primers, adaptors, and vectors using the Univec database (https://www.ncbi.nlm.nih.gov/tools/vecscreen/univec/).

Clean reads of each sample were mapped to the *A. thaliana* genome (TAIR 10.1—GCA_000001735.4) using STAR (Dobin et al., 2013), and the mapped reads were *de novo* assembled and quantified using StringTie (Kovaka et al., 2019). Novel transcripts were named as “MSTRG” by StringTie. TransDecoder package (http://transdecoder.sourceforge.net/) was used in the evaluation of transcript coding potential. We aligned all transcripts against NCBI-nr and Uniprot/UniRef90 (The UniProt Consortium, 2017) using BLASTx with an e-value cutoff of 1^e−3^. Once a sequence was identified as a potential coding one, it was excluded from the subsequent ncRNA analysis. We downloaded miRNA precursors from Rfam (Kalvari et al., 2018) in order to identify lncRNAs acting as miRNA precursors. Putative lncRNAs were also aligned to miRbase (Kozomara and Griffiths-Jones, 2010) sequences using BLASTn, and those showing cutoff >90% identification accuracy and e-value < 1^e−1^ were identified as probable miRNA precursors. The putative miRNA precursors and conserved lncRNAs were separated and excluded from the remaining dataset to prevent elimination in the next steps. The mature form of miRNAs was identified by a manual curation in all the miRNA fasta sequences, and those containing 20–22 nucleotides in length were classified as mature (miR). To discriminate between lncRNAs and sRNA transcripts, we aligned all remaining transcripts against the RNAcentral database (https://rnacentral.org/). The remaining transcripts were classified as candidate *A. thaliana* lncRNAs. To identify putative transposon sequences in lncRNAs, known transposon sequences of *A. thaliana* from the TAIR10 database (https://www.arabidopsis.org/) were downloaded. Long noncoding RNA sequences were aligned to the *A. thaliana* known transposons, and only those matching with at least 90% identity and e-value < 1^e−1^ were selected and classified as probable transposon sequences.

Next, we sought for natural antisense transcripts (NAT). After the alignment of lncRNA and PCGs using BLASTn, those NAT sequences showing ≥90% identity and no gap region longer than 150 bp were classified as putative lncRNA–NAT pairs. Finally, LncTar software (Li et al., 2015) was employed to confirm the annealing potential of the BLAST-predicted pairs.

### Differential expression analysis

To identify differentially expressed (DE) ncRNAs among the different treatments of *A. thaliana* seedlings, we used the prepDE.py3 script present in StringTie to extract gene count information from the program output. The ncRNA gene counts were used as input in the DESeq2 package (Love et al., 2014) (Bioconductor/R). Independent comparisons between samples from 1) GSNO-treated *rhd6* vs. untreated *rhd6*, 2) IAA-treated *rhd6* vs. untreated *rhd6*, 3) GSNO-treated *rhd6* vs. IAA-treated *rhd6*, and 4) WS-2 vs. untreated *rhd6* were performed. Normalization was carried out by adjusting the data distribution according to a negative binomial distribution, followed by removing the contigs with a base mean < 5. The adjusted *p*-value for each gene was calculated using the BH method (Benjamini and Hochberg, 1995), and only those with FDR < 0.05 were considered significant differentially expressed genes (DEGs).

### RNA pair interaction evaluation and network analysis

With the purpose of predicting lncRNA and PCG interaction, we used LncTar software. A cutoff value of −0.13 was used for the normalized free energy (ndG), which reflects the relative stability of internal base pairs in the paired RNAs. For testing the potential interaction between sRNA–lncRNA and sRNA–PCG, we used the heuristic mode in IntaRNA for searching (Mann et al., 2017). The lncRNA–PCG interaction types were identified by FEELnc software (Wucher et al., 2017) using the FEELnc_classifier.pl module. The output.gft file from StringTie *de novo* assembly and *A. thaliana* genome reference.gtf were used in the analysis. The interaction types could be classified as genic (lncRNA overlaps a PCG from the reference genome) and intergenic (lncRNA does not overlap a coding region). In addition, the genic lncRNA interactions could be classified as the following subtypes: 1) containing (lncRNA contains the mRNA partner), 2) nested (lncRNA is contained in the mRNA partner), and 3) overlapping (lncRNA partially overlaps PCG partner).

A protein–protein interaction (PPI) network with the PCG targeted by DElncRNA was constructed in Cytoscape v. 3.8.2 (Shannon et al., 2003). Proteins were queried against the STRING (https://string-db.org) database for the identification of interactions among them. The proteins were represented as circles (nodes) and the interactions among them were represented as lines (edges). The constructed hubs were submitted to enrichment analysis in Cytoscape, focusing on cell wall and nitrogen metabolism-related proteins. In addition, co-expression networks were analyzed and generated in CoExpNetViz (Tzfadia et al., 2015), using DElncRNA and DE CPG read counts as input, and selecting DElncRNA as bait nodes and default parameters. The co-expression networks produced were visualized in CoExpNetViz plugin in Cytoscape. We also performed a gene set enrichment analysis (GSEA) aiming to identify ncRNA gene classes with common expression patterns. The analysis was carried out in GSEA software v.4.2.3 (Subramanian et al., 2007) using 1,000 permutations, “phenotype” as permutation type, and FDR < 0.25, as recommended in the manual.

## Results

### Transcriptome overview

In our previous study with root hair *rhd6* mutants of *A. thaliana*, the RNA-seq approach identified 32,841 protein-coding genes, of which 6,670 were differentially expressed in response to GSNO and IAA (Moro et al., 2017). The ncRNA identification and evaluation of their expression profiles in the *A. thaliana* null *rdh6* mutant were performed using the same 16 libraries constructed by Moro et al. (2017), with four replicates of each: IAA-treated *rhd6*, GSNO-treated *rhd6*, untreated *rhd6*, and untreated WS-2. Here, we generated a total of 685,859,082 processed and cleaned reads, of which 658,226,876 (96%) were mapped against the *A. thaliana* reference genome (Supplementary Table S1). Transcripts with the evidence of protein-coding potential were separated and classified as the coding section of transcriptome, previously analyzed by Moro et al. (2017). After the removal of putative protein-coding transcripts, we identified 4,323 ncRNAs in the transcriptome of *A. thaliana*. Therefore, rRNA and tRNA were filtered out from the analysis, yielding a total of 3,631 ncRNA transcripts. The ncRNA genes were distributed in lncRNAs (>200 bp), with 2,768 being lncRNAs (125 novel) and 64 natural antisense transcripts (NAT). Among ncRNA <200 bp were 170 microRNAs (miRNAs) (77 presenting the mature form), 255 small nucleolar RNAs (snoRNAs), and 100 small RNA (sRNAs). The 100 sRNAs could not be classified neither in miRNA nor in snoRNA after annotation, remaining as sRNAs. We also identified 244 ncRNAs already known in *A. thaliana* genome, which could not be classified, being named as “other RNA” and 14 pseudogenes after RNA central and Rfam queries (Supplementary Table S2). Among the lncRNAs and pseudogenes, 85 and seven sequences are transposons, respectively.

**FIGURE 1:**
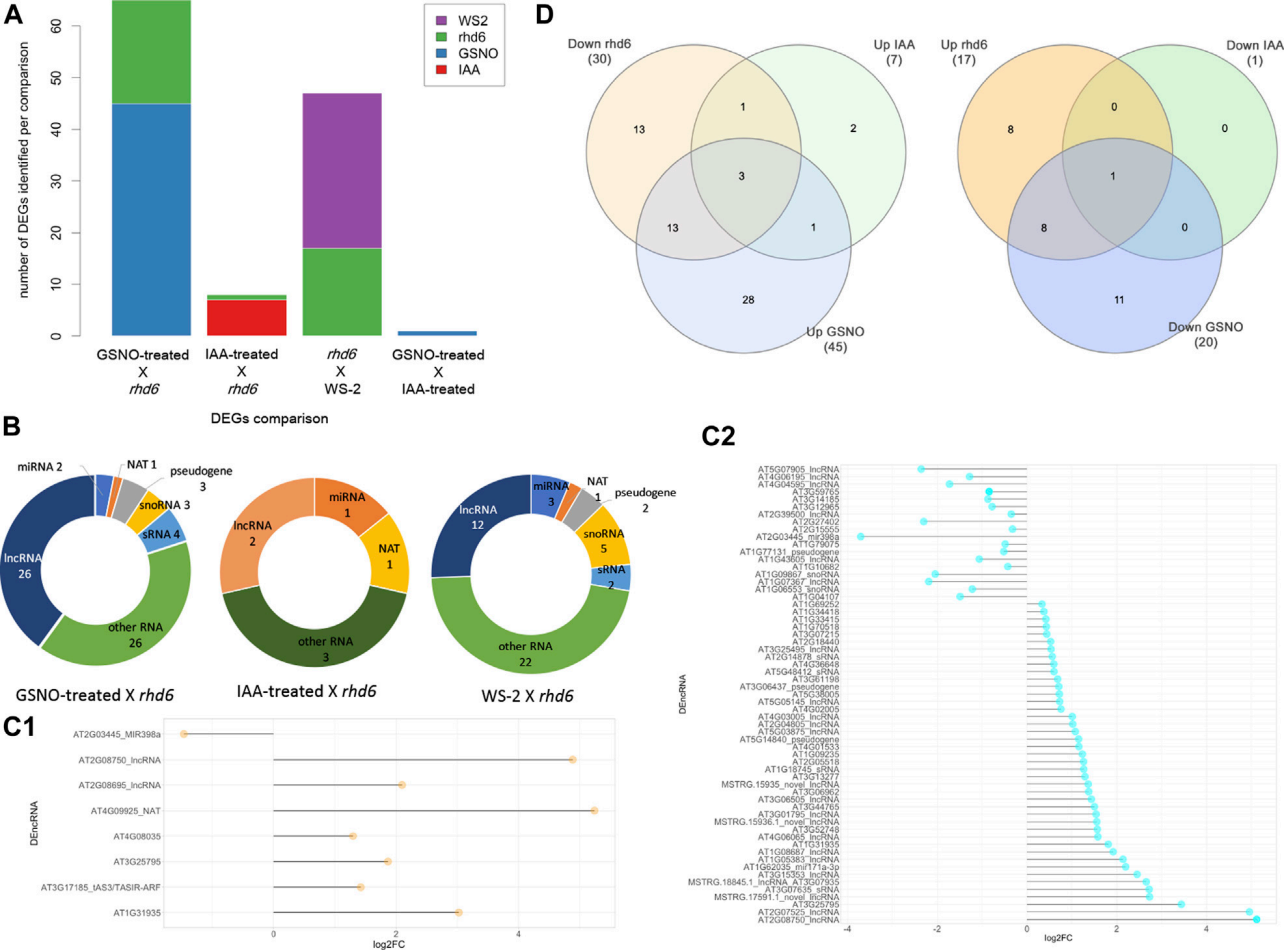
Differentially expressed gene (DEG) distribution of the four independent and paired comparisons performed. **(A)** Number of upregulated DEGs identified in the comparisons: GSNO-treated *rhd6* vs. untreated *rhd6*, IAA-treated *rhd6* vs. untreated *rhd6*, GSNO-treated *rhd6* vs. IAA-treated *rhd6*, and WS-2 vs. untreated *rhd6* (FDR < 0.05). **(B)** Noncoding RNA type distribution in each comparison. GSNO-treated *rhd6* vs. IAA-treated *rhd6* comparison is not shown due to the few DEGs found. **(C1)** Expression values detected for DEGs in IAA-treated *rhd6* vs. untreated *rhd6* and **(C2)** GSNO-treated *rhd6* vs. untreated *rhd6* comparisons. The *x*-axis shows the log2FC values, and positive values for upregulated and negative values for downregulated for IAA and GSNO treatments, respectively. The *y*-axis shows the DEGs names. **(D)** Left: union of DEGs identified downregulated in untreated *rhd6* mutants, and upregulated in IAA and GSNO treatments, revealing genes activated by each treatment. Right: union of DEGs identified upregulated in untreated *rhd6* mutants, and downregulated in IAA and GSNO treatments, revealing genes deactivated by each treatment.

We used all the ncRNA genes as a background reference for the differential expression analysis. Four independent and paired comparisons were performed between the following groups of *A. thaliana* seedlings: 1) GSNO-treated *rhd6* vs. untreated *rhd6*, 2) IAA-treated *rhd6* vs. untreated *rhd6*, 3) GSNO-treated *rhd6* vs. IAA-treated *rhd6*, and 4) WS-2 vs. untreated *rhd6* (FDR < 0.05) (Supplementary Table S3). The GSNO-treated *rhd6* showed the greatest number of differentially expressed genes (DEGs) (Figure 1A), with 45 upregulated ncRNA genes. From these, 39 had their ncRNA type identified after annotation, mostly being lncRNAs (Figure 1B). The expression values (log2FC) varied widely in IAA and GSNO-treated *rhd6* (Figures 1C1,C2). Among the 30 ncRNA genes downregulated between WS-2 and untreated *rhd6* seedlings, 13 are upregulated by GSNO, one by IAA, and three by both treatments (Figure 1D—left). On the other hand, considering the 17 DEGs upregulated between WS-2 and untreated *rhd6*, eight are downregulated by GSNO and one by both compounds, while no DEGs were detected to be downregulated only by IAA (Figure 1D—right).

### Long noncoding RNA interaction with protein-coding genes upon GSNO and IAA treatments

**FIGURE 2:**
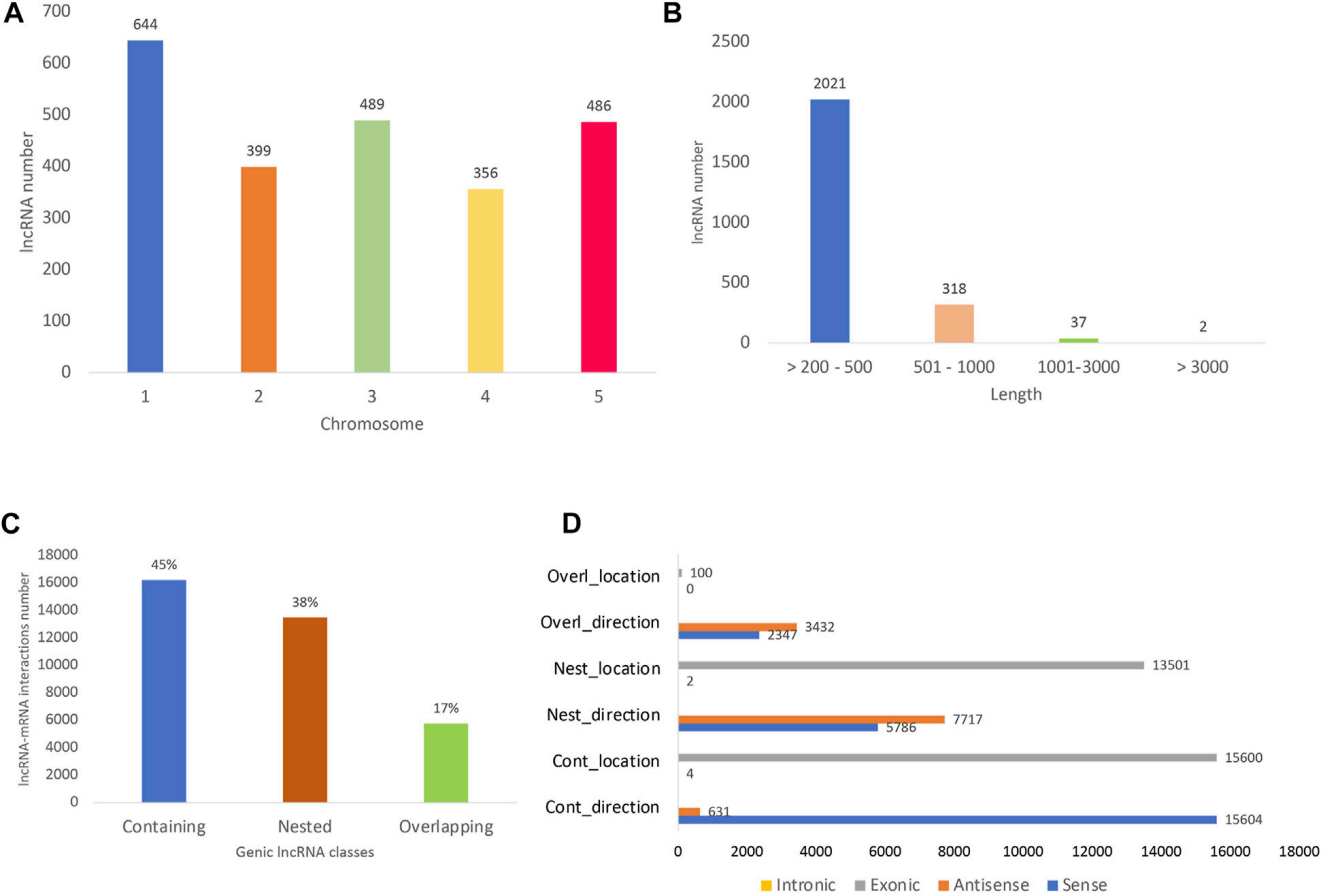
Long noncoding RNA (lncRNA) distribution. **(A)** Number of long noncoding RNAs already known (with AT identifiers) along the five *A. thaliana* chromosomes. **(B)** Length of all lncRNA identified, including those with AT identifiers and novel ones (the isoforms were not considered in the count). **(C)** Distribution of lncRNA and their respective differentially expressed protein-coding gene (PCG) targets. The interacting lncRNA–DEmRNA pairs were located mostly in genic regions (99.8%) and distributed in the following subtypes: containing (lncRNA contains the mRNA partner), nested (lncRNA is contained in the mRNA partner), and overlapping (lncRNA partially overlaps PCG partner). **(D)** lncRNA–PCG interaction direction and location, being mostly in sense direction and located in exonic regions. The *x*-axis shows the number of interactions detected and the *y*-axis shows the interaction direction and location. The number of interactions detected as intronic are very low compared to exonic ones, reason why no yellow bars could be observed for intronic interactions in the figure.

All the differentially expressed protein-coding genes (DE PCG) mentioned in this study were obtained from the same transcriptome analyzed here and previously reported by Moro et al. (2017). The lncRNA identified here are widely distributed in *A. thaliana* 1–5 chromosomes, with the greatest number found in chromosome 1 (27%) (Figure 2A). Long noncoding RNAs showed an average length of 274 bp, mostly ranging from 200 to 500 bp (85%) (Figure 2B). In terms of lncRNA and PCG interaction, we detected almost only genic interactions (99.8%). The genic lncRNA interactions identified were classified as the following subtypes: containing (45%), nested (38%), and overlapping (17%) (Figure 2C). Here, we identified the lncRNA–mRNA interactions mostly in sense direction (Figure 2D) and located in exonic regions.

**FIGURE 3:**
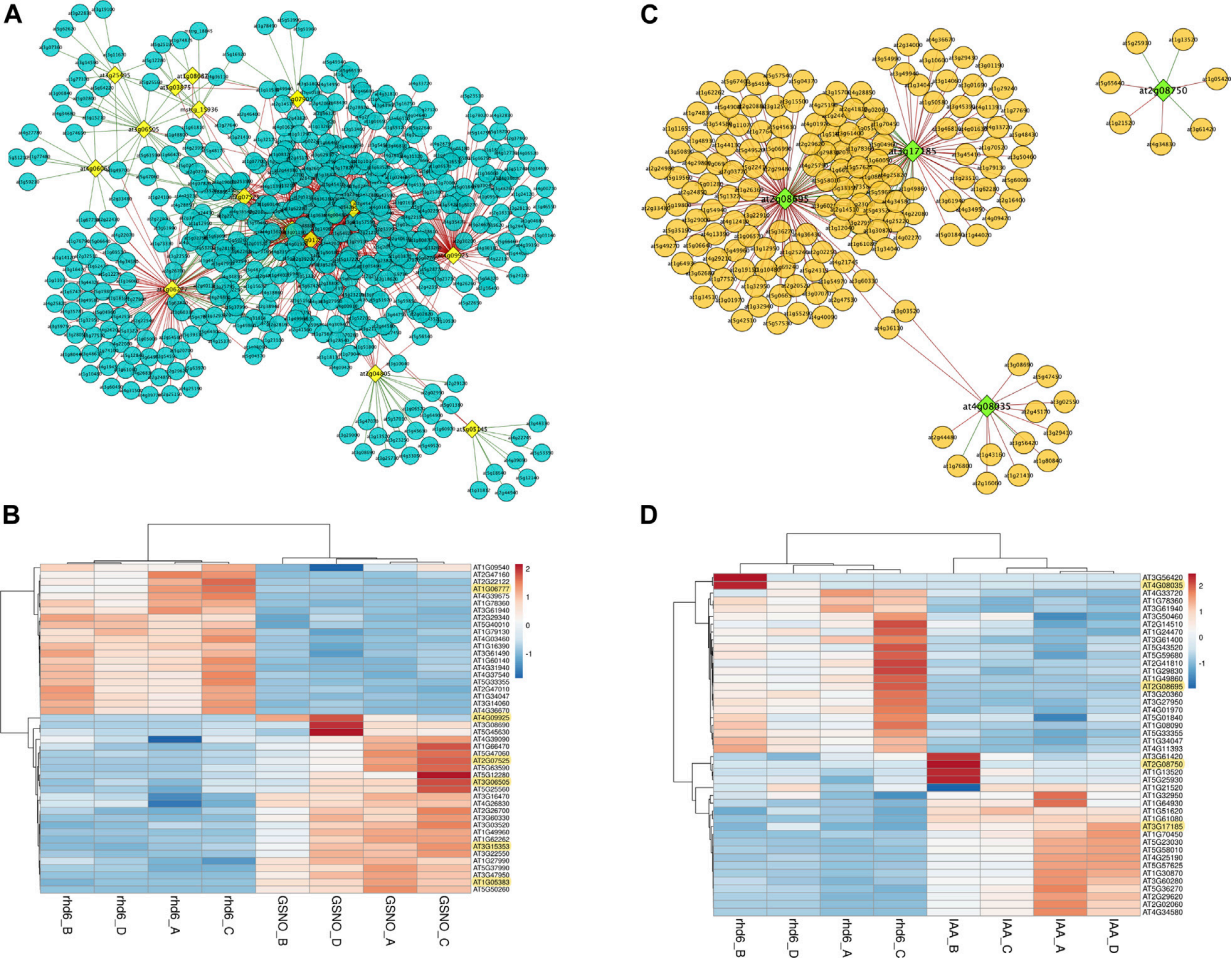
Results from co-expression analysis between differentially expressed (DE) long noncoding RNAs (DElncRNA) and differentially expressed protein-coding genes (PCGs). **(A)** Co-expression network generated for DElncRNA and DE PCGs which present similar expression patterns for GSNO treatment. The DElncRNA are represented in yellow and the DE PCG in blue. The lines represent the connections (edges) among DElncRNA and DE PCGs pairs, which may be correlated (green) or anticorrelated (red). **(B)** Heatmap showing the top 10 most correlated and anticorrelated PCGs and how they vary their expression patterns in the same or opposite direction to DElncRNAs, which are highlighted in yellow. All DElncRNA in the figure are correlated with DE PCG, except for AT1G06777. **(C)** IAA treatment co-expression network. The DElncRNA are represented in green and the DE PCG are represented in orange. Green lines represent correlated gene pairs, while red lines are anticorrelated gene pairs. **(D)** Top 10 heatmap with the most correlated and anticorrelated PCGs, and DElncRNAs are highlighted in yellow. AT2G08695 and AT4G08035 DElncRNAs are anticorrelated, while DEmRNA AT3G17185 and AT2G08750 are correlated. In Figures **(B,D)**, the respective color keys in the right side represent the z-scores.

We sought for interaction between the DElncRNA (lncRNA differentially expressed) identified in this work and the DE PCG described by Moro et al. (2017). Considering all the DElncRNA genes identified here and their respective PCG targets, 34 DE PCGs participate in biological processes related to the regulation of the nitrogen compound metabolic process. *WRKY40*, *SZF1*, *MYC2*, *WRKY48*, *AIB*, *MYB48*, and *NAC102* are potential candidate key genes in GSNO signaling pathways, under the modulation of DElncRNA identified here and usually interacting among each other (Supplementary Table S4). In addition, *PRP3*, *EXT13*, *RHS19*, and *MOP10* are included in the other set of 34 PCGs mostly involved in cell wall organization, biogenesis, and degradation (Supplementary Figure S1). We also analyzed how the expression pattern of DElncRNA identified here and DE PCGs varied under GSNO and IAA treatments, using the co-expression network analysis. Genes in a co-expression network may be positively (expression profile among DElncRNAs and DE PCGs rise or fall together among samples) or negatively (expression profiles vary in opposite directions among samples) correlated. Considering GSNO treatment, we detected 459 DE PCGs interacting with 16 key DElncRNA (Figure 3A; Supplementary Table S5). Although the connections among DElncRNA and DE PCGs are mostly negatively correlated, we observed some DElncRNA positively interacting with DE PCGs, such as AT3G25495, AT4G06065, AT2G04805, and AT5G05145 (Figure 3A). Differentially expressed lncRNA genes, AT1G06777, AT4G09925 (MIR5658 precursor), AT3G07525, AT3G06505, AT3G15353, and AT1G05383, are those with the strongest correlation values, with only AT1G06777 being negatively correlated (Figure 3B).

On the other hand, regarding IAA treatment, our analysis showed a total of 164 DE PCGs following the expression profiles of four main DElncRNA genes, highlighting AT2G08695 and AT3G17185 as those with the greatest number of connecting genes in the network (Figure 3C; Supplementary Table S5). AT2G08695 and AT4G08035 are the main DElncRNAs negatively correlated with DE PCGs, while AT3G17185 and AT2G08750 are positively correlated (Figure 3D). We observed 117 and 165 exclusive DE PCGs fluctuating their expression profiles according to DElncRNAs in GSNO and IAA treatments, respectively. Many biological roles such as development, hormone signaling, and protein modification were among those identified in DE PCGs for both treatments, highlighting the transcription factor group as the most represented one. A total of 11 and 29 cell wall-related genes were also detected as modulated by DElncRNA for GSNO and IAA treatments, respectively (Supplementary Table S5).

We also identified some ncRNA >200 bp that could not be annotated as lncRNA, although with important roles in the NO metabolism. After LncTar analysis, which calculates the minimum free energy joint structure of two RNA molecules based on base pairing, we identified the ncRNA AT3G25795 and the novel MSTRG.17591 as key DEncRNAs in GSNO treatment. AT3G25795 appears upregulated in the GSNO-treated and WS-2 seedlings (average log2FC = 3.62), and interacting with DE PCGs related to root hair formation (*EXT13*) and transmembrane protein (*MUL8*), both upregulated in GSNO-treated seedlings. Novel MSTRG.17591 also is upregulated in GSNO-treated and WS-2 individuals (average log2FC = 2.69), targeting the PCG involved in protein degradation (*ATG8E*), which is induced in GSNO-treated and inhibited in IAA-treated seedlings (Supplementary Table S6). In addition, we also detected the long noncoding RNA AT4G09925 (MIR5658 precursor), highly upregulated in GSNO-treated and WS-2 seedlings (log2FC = 4.88 and 5.02, respectively) and downregulated in IAA-treated seedlings (log2FC = −5.23). Furthermore, we identified a total of 64 NATs in *A. thaliana* transcriptome expressed under GSNO and IAA treatments, along with 13 lncRNA–NAT, not differentially expressed, overlapping with DE PCG with biological roles related to development, transcription factors, calcium regulation, protein degradation, heat shock protein, and root hair formation (Supplementary Table S2).

### miRNA interaction with lncRNA and protein-coding genes on GSNO- and IAA-treated seedlings

**FIGURE 4:**
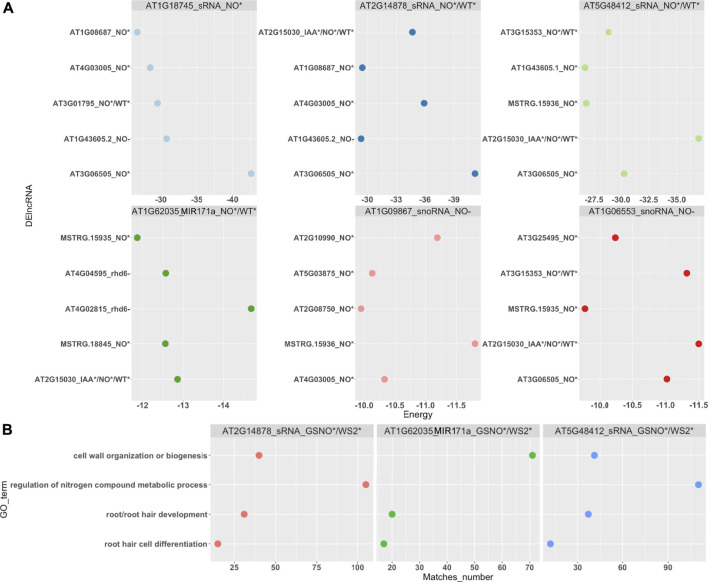
Small RNA (sRNA) interaction with DElncRNAs and DE PCG. As sRNA, we consider sRNA (could not have their types identified by annotation), nucleolar RNA (snoRNA), and microRNA (miRNA). **(A)** Top six DElncRNA–sRNA pairs with the strongest interaction energy (negative values). Three of them are differentially expressed in GSNO-treated and WS-2 individuals, potentially acting in the WS-2 root hair formation WS-2 phenotype. The *x*-axis shows the interaction energy values, whereas the *y*-axis represents the DElncRNA genes and their differential expression condition. **(B)** sRNA and DE PCG interaction. Top three DE PCG with the greatest number of Gene Ontology (GO) biological process (BP) terms matches. The BPs are mostly related to nitrogen metabolism and cell wall organization and root development. The *x*-axis represents the BP matches number and the *y*-axis represents the most common BP terms identified.

We also sought for ncRNA <200 bp or sRNA, such as miRNA and snoRNA. These RNAs are known to target other ncRNAs, such as lncRNAs and also PCGs. Here, we identified 15 differentially expressed sRNA, mostly under GSNO treatment in the *rhd6* mutant, such as the microRNAs MIR171 and miR398 (Supplementary Table S3). We investigated the interaction between DEsRNA and DElncRNA, in which six DEsRNA with the highest interaction energies were all differentially expressed under GSNO treatment and also targeting DElncRNA responsive to GSNO treatment (Figure 4A; Supplementary Table S6). Three DEsRNA, AT2G14878 (sRNA), AT5G48412 (sRNA), and AT1G62035 (MIR171), are upregulated in GSNO-treated *rhd6* and WS-2, and are potentially involved in the root hair phenotype restoration in null *rhd6* mutants.

In addition, we evaluated the interaction between DEsRNA and DE PCG, selecting those loci with the highest interaction energies (<−20 kcal/mol) and a greater number of PCG targets. Here, we highlight one more time AT2G14878, AT5G48412 and AT1G62035 (MIR171), all responsive in GSNO-treated *rhd6* and WS-2 seedlings. Their respective targeted PCGs show, among the top GO terms identified for biological processes (BP), cell wall organization, root hair development/differentiation, and regulation of nitrogen compound metabolic process (Figure 4B). On the other hand, we detected TAS3 (AT3G17185), a small interference RNA (siRNA), upregulated in IAA-treated *rhd6* and interacting with small auxin upregulated RNA 6 (*SAUR6*) (Supplementary Tables S3, S6).

### Nitric oxide central importance in the restoration of the wild root hair phenotype in *A. thaliana*

**FIGURE 5:**
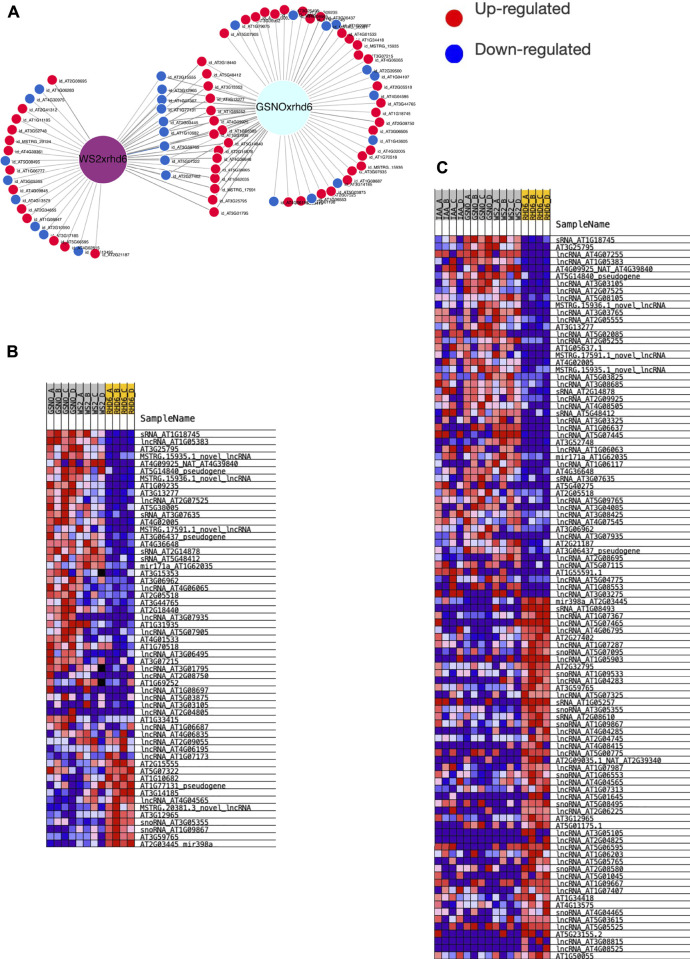
Differentially expressed genes in GSNO-treated and WS-2 seedlings and their potential participation in the restoration of root hair phenotype in *rhd6* mutants. **(A)** Intersection of differentially expressed genes (DEGs) detected in the comparison between GSNO-treated *rhd6* vs. untreated *rhd6* and WS-2 vs. *rhd6* reveals 25 genes in common for GSNO-treated *rhd6* and WS-2 seedlings, of which nine are downregulated and 16 are upregulated. **(B)** Gene set enrichment analysis (GSEA) performed for GSNO-treated *rhd6*/WS-2 vs. untreated *rhd6* groups. Figure shows DE genes are enriched and contribute with similar expression patterns for the WS-2 root hair formation (RHF) phenotype. **(C)** GSEA performed for GSNO-treated/IAA-treated/WS-2 vs. *rhd6* groups. Figure shows the top 50 genes contributing the most in GSNO- and IAA-treated for the recovery of RHF phenotype observed in WS-2.

Based on the determinant role of NO in *A. thaliana* root hair formation and in the restoration of wild root hair phenotype (Moro et al., 2017), we investigated which types of DEncRNA, in general, may potentially be involved in this process. For this purpose, we overlapped the DEGs identified in GSNO-treated *rhd6* vs. untreated *rhd6* and WS-2 vs. untreated *rhd6*, resulting in 25 common DEGs for the two comparisons, nine being downregulated and 16 upregulated for GSNO-treated and WS-2 seedlings (Figure 5A). Aiming to identify genes with similar expression patterns in recovering the wild-type root hair phenotype in the *rhd6* mutant, we used GSEA (FDR < 0.25). First, we analyzed the similar expression pattern between GSNO-treated *rhd6*/WS-2 vs. untreated *rhd6* groups DEGs, identifying 36 out of 45 GSNO-treated upregulated genes among the enriched ones (Figure 5B). Our results suggest some DEncRNAs that contribute to restore the root hair phenotype of the hairless *rhd6* mutant. In addition, we swept the whole ncRNA transcriptome, considering differentially expressed genes and those in which differential expression was not detected. The GSEA was employed to compare the groups of GSNO-treated *rhd6*/IAA-treated *rhd6*/WS-2 vs. untreated *rhd6* (Figure 5C). Therefore, a new set of genes potentially contributing to root hair phenotype restoration in GSNO-treated and IAA-treated *rhd6* seedlings were revealed, bringing 10 upregulated and two downregulated genes detected in GSNO-treated seedlings (Supplementary Table S7). As shown in Figures 5B,C, MIR171 and MIR5658 precursors, and the novel lncRNAs MSTRG 15935, 15936, and 17591 are listed as key DE ncRNAs involved in the root hair phenotype restoration in *A. thaliana rhd6* mutants.

## Discussion

With the purpose of identifying ncRNAs and assessing their expression profiles in the *A. thaliana* null *rdh6* mutant, we used the same 16 libraries constructed by Moro et al. (2017), four of each abovementioned condition. We addressed the lncRNA genes expressed in *rhd6 A. thaliana* root seedlings, aiming to verify which lncRNAs were activated (upregulated) or deactivated (downregulated), and how GSNO and IAA exposure modulate the lncRNA and their respective mRNA target gene expression. In addition, we evaluated the putative interactions between ncRNA–ncRNA and ncRNA–PCG pairs upon GSNO and IAA treatments, in three major groups: 1) lncRNA and PCGs, 2) miRNA and lncRNA, and 3) miRNA and PCGs.

Among the 3,631 noncoding RNAs analyzed in the present study, we identified many ncRNA potentially involved in the restoration of the root hair phenotype in the *rhd6* mutant by GSNO. The co-expression network analysis between DElncRNA and DE PCG targets revealed close to 460 DE PCG interacting positively or negatively with 16 central DElncRNA upon GSNO treatment, being one of them not yet identified for *A. thaliana* (MSTRG 15936). Among the lncRNA interactions with positive values is MIR5658, a long noncoding NAT detected here upregulated in GSNO-treated and WS-2 seedlings, and downregulated in IAA-treated seedlings (Supplementary Table S3). MIR5658 has already been identified in different plant species as involved in plant development, hormone signaling, and tolerance to abiotic stress (Biniaz et al., 2022). This miRNA precursor directly upregulates the expression of AT3G25290, a member of auxin-responsive gene family, by targeting its promoter. This activation may be involved in the development and growth of *A. thaliana* (Yang et al., 2019). In our analysis, the MIR5658 precursor appears among the most differentially expressed lncRNAs, upregulated in GSNO-treated *rhd6* (Figure 3B), and seems to be an important regulator that represses the expression of numerous PCGs, but not AT3G25290 (Supplementary Table S5). This precursor is also known for controlling the expression of transcription factors, as those related to growth and development in *A. thaliana*, such as *GRAS* (gibberellic-acid insensitive) and *ERF* (ethylene responsive factor) (Rakhmetullina et al., 2021), mostly upregulated in the study by Moro et al. (2017). In addition, we identified some cell wall-related genes (xyloglucan endotransglucosylases, expansins, and arabinogalactans), whose expression levels varied along with DElncRNAs and were regulated by GSNO treatment (Supplementary Table S5). Xyloglucan endotransglucosylases can act in the degradation and loosening of cell wall, resulting in abnormal root hair formation and growth (Cavalier et al., 2008; Hayashi and Kaida, 2011). Moreover, arabinogalactans and expansins are closely involved in cell wall morphogenesis processes, as cell differentiation and cell wall expansion (Ellis et al., 2010; Lin et al., 2011). Furthermore, other 13 lncRNA–NAT not differentially expressed were detected, overlapping with DE PCG and acting in many biological roles, such as root hair formation (Supplementary Table S2). All of those PCGs are responsive to GSNO treatment and are mostly downregulated, such as WRKY transcription factor 61 (*WRKY61*), belonging to a protein family required for a myriad of biological events related to plant defense, stress, and development (Jiang et al., 2017; Singh et al., 2019).

In addition, we detected microRNAs MIR171 and miR398 differentially expressed in our transcriptome. According to Yan et al. (2022), the signaling pathway of MIR171 in root development is still unknown. Since this microRNA was upregulated in GSNO-treated roots with a similar WS-2 expression pattern, and considering its relevant role in root development (Yan et al., 2022), our data suggest that NO could be one of the signaling molecules implicated in restoring the root hair phenotype in *rhd6* mutant through MIR171 regulation. Among other biological processes, MIR171 regulates root hair differentiation, by targeting some protein-coding genes as those from the scarecrow-like family (SCL) and scarecrow (SCR) GRAS domain transcription factors (Singh et al., 2021). According to our present and previous data (Moro et al., 2017), MIR171 along with *SCL8* and *SCRL7* is upregulated in GSNO-treated *rhd6* seedlings. The interaction between DEsRNA and DElncRNA pairs were also tested under both treatments. Three DEsRNA, AT2G14878 (sRNA), AT5G48412 (sRNA), and AT1G62035 (MIR171) are upregulated in GSNO-treated *rhd6* and WS-2, and are potentially involved in the root hair phenotype restoration in null *rhd6* mutants. As observed in Figure 4, those DEsRNA target some novel DElncRNA for *A. thaliana*, upregulated in both GSNO-treated and WS-2 seedlings, indicating those pairs as candidates in the restoration of normal root hair formation in *rhd6* seedlings. When considering the interactions between DEsRNA and DE PCG pairs (energy < −20 kcal/mol), we again highlight the three DEsRNA abovementioned, all responsive in GSNO-treated *rhd6* and WS-2 seedlings, and involved in cell wall organization, root hair development, and regulation of the nitrogen metabolism. Interestingly, AT2G14878 was identified as one of the 2,006 genes producing mobile RNAs in *A. thaliana*, which are systemically delivered to distant tissues, being transported in both directions, from root to shoot and from shoot to root (Thieme et al., 2015).

Small interference RNA (siRNA) TAS3 (AT3G17185) was upregulated in IAA-treated *rhd6* and interacting with small auxin upregulated RNA 6 (*SAUR6*) (Supplementary Tables S3, S6). TAS3 is known to suppress gene expression by post-transcriptional gene silencing in plants, orchestrating lateral root (LR) formation in *A. thaliana* by the modulation of miR390, and auxin response factors (*ARF*), as part of the auxin-mediated molecular network (Marin et al., 2010; Meng et al., 2010; Yoon et al., 2010). Yoon et al. (2010) shed light on the role of the miR390/TAS3/ARF pathway in the detection of auxin concentration and LR development. In our previous study, we identified *ARF9*, *ARF16*, and *ARF17* upregulated in IAA- and GSNO-treated *rhd6* seedlings (Moro et al., 2017), agreeing with our recent detection of TAS3 induced upon IAA treatment. Another important miRNA acting in root development is miR160 (Liang et al., 2012), also modulating the expression of *ARF16* and *ARF17*, which were upregulated in GSNO-treated *rhd6* seedlings in the study by Moro et al. (2017).

According to our findings (Figures 5B,C), MIR5658 and MIR171 precursors were upregulated in GSNO-treated *rhd6* and WS-2 seedlings. Along with the novel lncRNAs MSTRG 15935, 15936, and 17591, they are the key ncRNAs interacting with DE PCGs to restore the wild-type root hair phenotype. A much clearer and more determinant influence of GSNO was observed in the *A. thaliana* root hair noncoding transcriptome when compared to IAA, which is in line with previous PCG data shown in the study by Moro et al. (2017).

## Data Availability

The datasets presented in this study can be found in online repositories. The names of the repository/repositories and accession number(s) can be found below: https://www.ncbi.nlm.nih.gov/, SRP285694.

## Supplementary material

SUPPLEMENTARY FIGURE S1 Long noncoding PCG target genes and how their gene products interact among each other in a protein–protein interaction (PPI) network. The DE PCGs with red and green circles are those GO enriched and with gene products related to nitrogen metabolism and cell wall organization, respectively.

SUPPLEMENTARY TABLE S1
*A. thaliana* transcriptome overview. Number of raw reads generated, reads processed, and cleaned and reads remaining after mapping against thale cress reference transcriptome.

SUPPLEMENTARY TABLE S2 Noncoding RNA (ncRNA) types identified in *A. thaliana* transcriptome. The ncRNA types are distributed in long noncoding RNA (lncRNA), longer than > 200 bp; natural antisense transcripts (NATs); small RNA (sRNA), including sRNA (no type could be identified by annotation), micro-RNA (miRNA), and nucleolar RNA (snoRNA); and other RNA which noncoding type could not be identified by annotation. The loci containing transposon sequences are also shown.

SUPPLEMENTARY TABLE S3 Differentially expressed genes (DEGs) identified in the four independent and paired comparisons performed: GSNO-treated *rhd6* vs. untreated *rhd6*, IAA-treated *rhd6* vs. untreated *rhd6*, GSNO-treated *rhd6* vs. IAA-treated *rhd6*, and WS-2 vs. untreated *rhd6* (FDR < 0.05). Upregulated and downregulated genes for each condition are shown, along with log2FC and false discovery rate (FDR) values.

SUPPLEMENTARY TABLE S4 Results from long noncoding PCG target genes GO enrichment performed in STRING database. Some of the nitrogen metabolism related genes (red circles from Supplementary Figure S1) shown in protein–protein interaction (PPI) network are highlighted in yellow.

SUPPLEMENTARY TABLE S5 Results from the co-expression analysis performed among differentially expressed genes long noncoding RNA (DElncRNAs) and differentially expressed protein-coding genes (DE PCGs) for GSNO- and IAA-treated seedlings. The edge sheets show gene pairs correlation (positive values) or anticorrelation (negative values). The node sheets show DElncRNA (bait node) and DE PCG (family node) identification. Additionally, the list of exclusive DE PCG identified for GSNO and IAA treatments are provided along with their biological role.

SUPPLEMENTARY TABLE S6 Details from 1) DElncRNA and DEmRNA, 2) DEsRNA and DElncRNA, and 3) DEsRNA and DEmRNA interactions analysis performed. Stronger interactions are represented by smaller negative values (kcal/mol).

SUPPLEMENTARY TABLE S7 Gene set enrichment analysis (GSEA) results for 1) GSNO-treated/WS-2 vs. IAA-treated/untreated *rhd6* and 2) GSNO/IAA-treated/WS-2 vs. untreated *rhd6* groups. The enriched genes are shown and those upregulated for GSNO are highlighted in yellow, whereas those downregulated are highlighted in green.
