# Supplementary material for: Noncoding RNAs responsive to nitric oxide and their protein-coding gene targets shed light on root hair formation in Arabidopsis thaliana
Source: Front Genet. 2022 Sep 27;13:958641. doi: 10.3389/fgene.2022.958641 (PMC9551039; doi:10.3389/fgene.2022.958641)
Supplement: Supplementary file 1 [file DataSheet1.zip › Data Sheet 1/TableS1.docx]

| **Illumina reads**  ***A. thaliana* transcriptome *de novo* assembly and mapping overview** | | | | |
| --- | --- | --- | --- | --- |
| **Libraries (raw data)** | | | | |
| **Treatment** | **IAA**  **(4 libraries)** | **GSNO**  **(4 libraries)** | **rhd6**  **(4 libraries)** | **WS-2**  **(4 libraries)** |
| Paired-end reads | 227,437,772 | 194,600,190 | 228,920,900 | 244,766,092 |
| Average paired-end reads/ library (millions) | 56,8 | 48,6 | 57,2 | 61,2 |
| Standard deviation | 7,726,179 | 7,510,784 | 9,142,408 | 1,569,750 |
| **Total** | **895,724,954** | | | |
| **After SeqyClean filtering** | | | | |
| Paired-end reads | 170,877,078 | 148,174,784 | 173,128,084 | 193,679,136 |
| Average paired-end reads/ library (millions) | 42,7 | 37,0 | 43,2 | 48,4 |
| Standard deviation | 5,825,384 | 4,764,579 | 7,073,511 | 2,745,144 |
| **Total** | **685,859,082 (76.5%)** | | | |
| **After mapping** | | | | |
| Paired-end reads | 165,073,686 | 140,901,416 | 166,855,436 | 185,396,338 |
| **Total mapped** | **658,226,876 (96%)** | | | |
