# Supplementary figures and images for: Noncoding RNAs responsive to nitric oxide and their protein-coding gene targets shed light on root hair formation in Arabidopsis thaliana
Source: Front Genet. 2022 Sep 27;13:958641. doi: 10.3389/fgene.2022.958641 (PMC9551039; doi:10.3389/fgene.2022.958641)

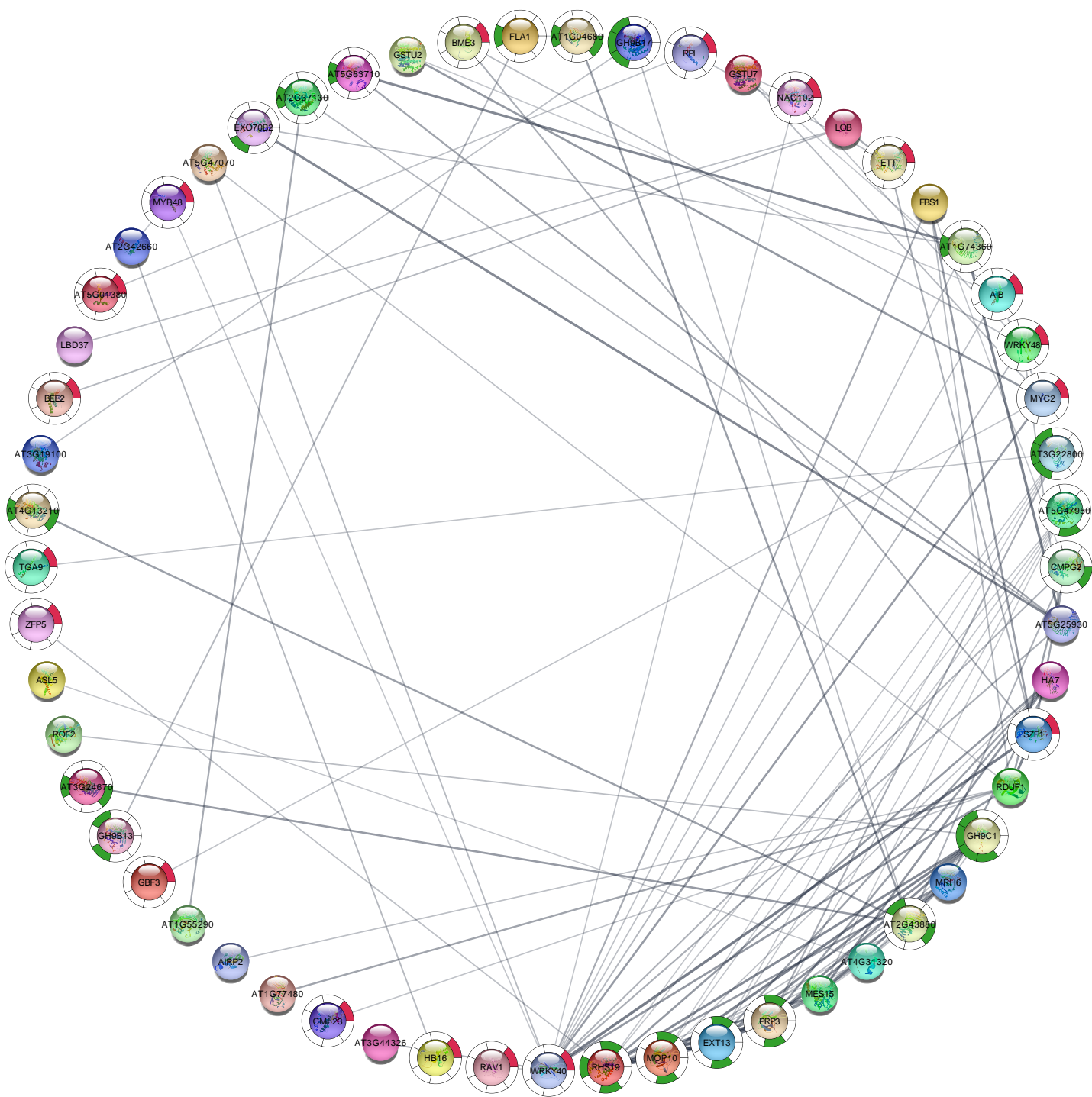

Supplement: Supplementary file 1 [file DataSheet1.zip › Data Sheet 1/FigureS1.pdf]
